# Role of gut symbionts of insect pests: A novel target for insect-pest control

**DOI:** 10.3389/fmicb.2023.1146390

**Published:** 2023-03-13

**Authors:** Pravara S. Rupawate, Praveen Roylawar, Kiran Khandagale, Suresh Gawande, Avinash B. Ade, Durgesh Kumar Jaiswal, Seema Borgave

**Affiliations:** ^1^Department of Zoology, Sangamner Nagarpalika Arts, D. J. Malpani Commerce and B. N. Sarda Science College (Autonomous), Sangamner, Maharashtra, India; ^2^Department of Botany, Sangamner Nagarpalika Arts, D. J. Malpani Commerce and B. N. Sarda Science College (Autonomous), Sangamner, Maharashtra, India; ^3^ICAR-Directorate of Onion and Garlic Research, Pune, India; ^4^Department of Botany, Savitribai Phule Pune University, Pune, India

**Keywords:** host–microbe interaction, immunity, microbial detoxification, nutrition, parasitoid, pest control, plant defence, symbiont-mediated

## Abstract

Insects possess beneficial and nuisance values in the context of the agricultural sector and human life around them. An ensemble of gut symbionts assists insects to adapt to diverse and extreme environments and to occupy every available niche on earth. Microbial symbiosis helps host insects by supplementing necessary diet elements, providing protection from predators and parasitoids through camouflage, modulation of signaling pathway to attain homeostasis and to trigger immunity against pathogens, hijacking plant pathways to circumvent plant defence, acquiring the capability to degrade chemical pesticides, and degradation of harmful pesticides. Therefore, a microbial protection strategy can lead to overpopulation of insect pests, which can drastically reduce crop yield. Some studies have demonstrated increased insect mortality *via* the destruction of insect gut symbionts; through the use of antibiotics. The review summarizes various roles played by the gut microbiota of insect pests and some studies that have been conducted on pest control by targeting the symbionts. Manipulation or exploitation of the gut symbionts alters the growth and population of the host insects and is consequently a potential target for the development of better pest control strategies. Methods such as modulation of gut symbionts *via* CRISPR/Cas9, RNAi and the combining of IIT and SIT to increase the insect mortality are further discussed. In the ongoing insect pest management scenario, gut symbionts are proving to be the reliable, eco-friendly and novel approach in the integrated pest management.

## 1. Introduction

Insects form a highly adaptable and abundant group of animals observed in diverse habitats (Basset et al., 2012). In tracing the evolutionary path of the class insecta, bugs and butterflies are the first one to conquer the earth (Misof et al., 2014). The successful evolution of insects by addressing and overcoming numerous environmental challenges was made possible, likely due to their close association with gut symbionts. The gut symbionts help host insects meet their needs for essential diet elements, protection from biotic interaction partners such as plants, pathogens, and predators, communication by optimizing the release of pheromones, etc. (Gupta and Nair, 2020). Insects, which have emerged as a highly successful clade, are the biological foundation for variety of ecosystems and they play a key role in shaping every ecological niche. They perform most of the pollination resulting in better plant productivity (Engel and Moran, 2013).

Although they play an important role in plant pollination, some insects become a nuisance after gaining pest status. There is a significant loss in the agricultural sector when the population of these insect pests rises beyond control. The FAO (Food and Agriculture Organization of the United States) has reported losses of about 40% losses of the world’s total crops each year due to the intervention of insect pests. Reports suggest that at least $70 million is spent to manage this infestation through various pest management strategies, which can also result in biodiversity loss. The Entomological Society of America has confirmed that spending ~$2.5 billion on pest control is associated with concomitant loss of $18 billion per year due to lost productivity in crops, lawns, forests, and pastures (The Not-So-Hidden Dangers of Invasive Species 2018). Plant pests affect agricultural activities and indirectly affect the income stream of rural communities. Various human activities, such as frequent relocations, global import and export of goods, climate change and changes in agricultural practices, have led to an increase in the population of invasive crop pests. At the same time, these activities are also responsible for an increase in urban pest populations and disease vectors (Nicolopoulou-Stamati et al., 2016).

Chemical insecticides are the most frequently and conventionally used approaches to control insect pests. However, due to increasing awareness of associated environmental issues, human health and biodiversity concerns, and increased pesticide tolerance by insects, there is a need to limit the use of chemical insecticides. In order to get rid of the side effects of chemical pest control methods, various microbial-based control strategies and tools are now emerging. Bacteria can be exploited to turn them into natural enemies against insect pests, e.g., *Photorhabdus luminescens*, entomopathogenic bacteria, control the population of the African migratory locust, *Locusta migratoria migratorioides* (Muhammad et al., 2022). Similarly, the population of the Brinjal shoots and fruit borer (BSFB), *Leucinodes orbonalis* Guenee, is controlled by the indigenous *Bacillus thuringiensis* strain VKK-BB2 (Pola et al., 2022). Furthermore, genetic manipulation of bacteria and feeding them to the pest insects may result in an effective pest control measure, as observed in *Bactrocera dorsalis,* the oriental fruit fly. When *Bactrocera dorsalis,* is fed genetically modified *E. coli*, high insect pests’ mortality has been observed (Mohanpuria et al., 2021). Studies encompassing the gut microbiomes of insects and their roles in insect physiology could be used to develop novel insect pest control tools. Insect gut microbiota have emerged as extremely useful tools for introducing biocontrol strategies. The introduction of modern technologies such as high throughput sequencing, various functional omics, gene editing, etc. has fuelled the discovery of novel bacteria in a rapid and accelerated manner. These newly discovered bacteria can be implemented using a variety of methods, which also help to unravel the precise role of these microbes in the complex biological systems of insects. Incompatible Insect Technique (IIT), RNAi mediated paratransgenesis, paratransgenesis, exploitation of chemical inventories of the microbiome to develop novel bio-pesticides, microbial semiochemicals, CRISPR/Cas9 system, a combination of bio-pesticides with nanotechnology, autocidal program, and sterile insect technique, are some of the modern techniques. The outcome of modern pest control methods is quite conclusive (Qadri et al., 2020). The present review focuses on the importance of gut symbionts in insect pests and possible ways to manipulate these symbionts to achieve eco-friendly and targeted pest management strategies.

## 2. Symbiotic microbes: A boon for insects

**Figure 1:**
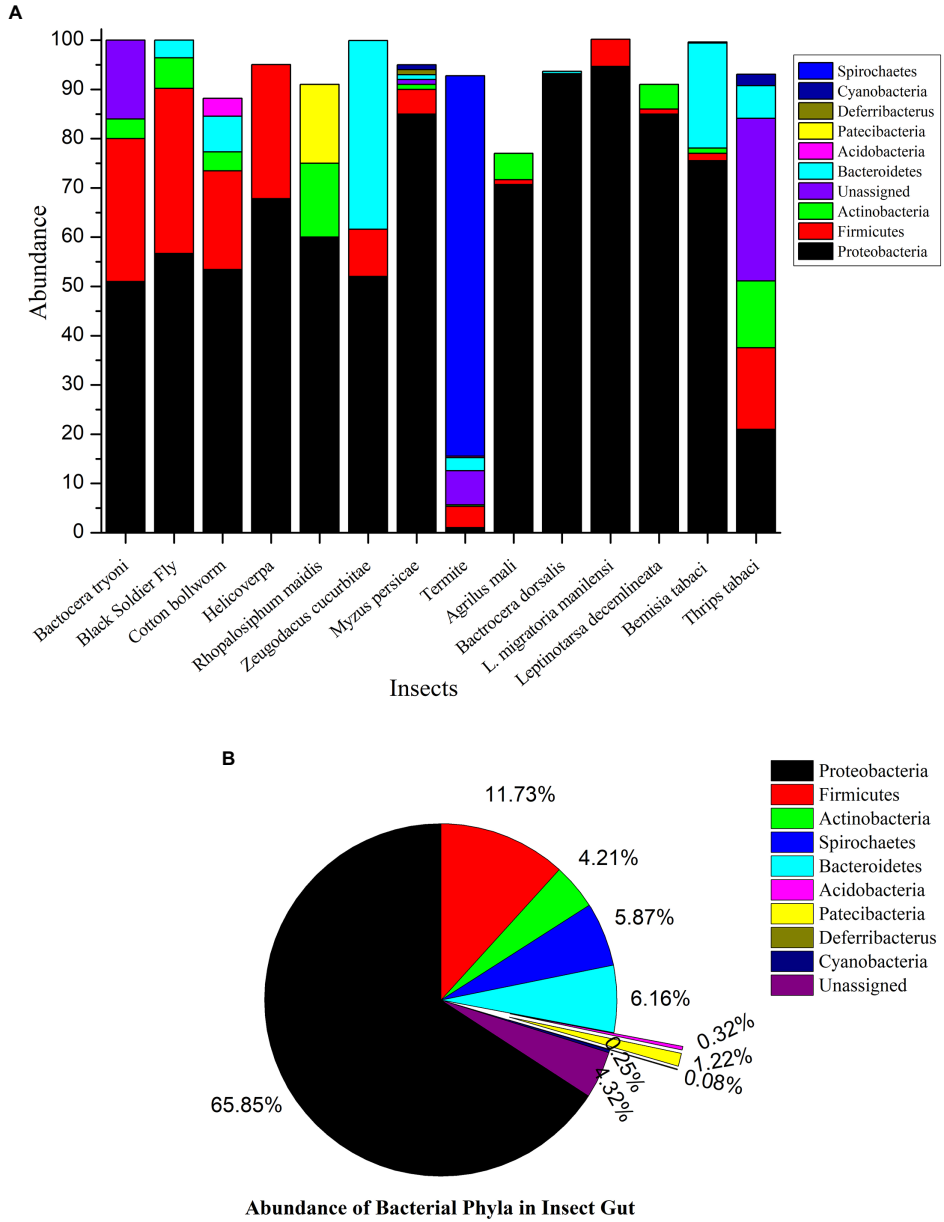
**(A)** Metagenomics study of various insect pests. 16s rRNA based metagenomics studies reveal that insect gut is resided by number of bacterial genera. **(B)** Microbial Diversity and abundance in the various insect pests. This figure illustrates the dominance of Proteobacteria among the other bacteria inside the insect gut. They perform variety of functions. Very few insect species have acquired bacteria from phylum Acidobacteria.

Microbial diversity is a large part of the world’s biodiversity. Microorganisms have maintained various types of interactions, including symbiosis with humans, animals, plants, etc. Microbes play myriad roles in the host metabolism; therefore, an animal’s overall metabolome is the sum of its genetic code plus the resident microbes (Douglas and Dobson, 2013). The acquisition of bacteria by insects *via* environment is the first route to establish the microbial symbiosis and subsequently, after attaining the entry these bacteria changes their free living status to symbionts. Such intracellular bacteria acquired by insects is rather common in nature and it ispredicted that nearly 15% of insects harbor such mutualistic microbes (Douglas, 1989). Insects provide special niche and a slot for these microbes and their existence strongly depends on physiological conditions and life cycle of host (Engel and Moran, 2013). Further, these endosymbionts harbored by insects, are classified as obligate symbionts, facultative symbionts and phytopathogenic symbionts, based on their localization, distinct features and function. Obligate mutualist usually transmits *via* vertical transfer and tends to occupy bacteriocytes, providing nutrition and fitness benefits to the insect hosts. Bacteriocytes are mostly present in proximity of digestive system (epithelium of midgut) to ease in transfer of nutrients. These symbionts have very stable accommodation in hemipteran sap sucking insects where diet is devoid of essential nutrients. In aphids, the obligate symbiont *Buchnera aphidicola* accomplish the nutritional need by synthesizing rare vitamins and essential amino acids (Feng et al., 2019). Contrary to obligate symbionts, facultative symbionts are transmitted either vertically, e.g., *Wolbachia,*or acquired at every generation *via* environment, e.g., *Burkholderia* and get acquired not only in bacteriocytes but in haemocoel or confined to other systems of host insects. Facultative symbionts help host insects in pesticide detoxification, e.g., the symbiont *Pseudomonas* assist the host insect to get rid of ingested pesticides (Almeida et al., 2017). Despite having beneficial role in insect body, facultative symbionts turn into commensals or pathogenic under certain circumstances (Marubayashi et al., 2014). Further, the phytopathogenic symbionts resides in insect body and they are dependent on insect vector for their transmission toward plants. These symbionts actively involve in interaction with insect hosts and facilitate insects in feeding. *Xylella fastidiosa* is the phytopathogenic symbiont residing in the foregut of leafhoppers and spittlebugs (Chatterjee et al., 2008). Detailed mechanism of host and phytopathogenic symbiont interaction is not discussed in this review as it is beyond the scope of subject of this review. Over many decades of evolution, gut microbial symbionts have been observed to be an integral part of most insects. Most of these symbionts are confined to gut epithelium due to favorable physiological conditions in the gut; metagenomics approach uses insect gut as tissue sample for analysis, however for tiny insects, due to their size, whole insect is treated as sample for further experiments. To decode the functional implications of these gut symbionts, it is necessary to study their distribution. Thus, we have summarized recent metagenomics studies (whole insect and insect gut) of several insect pests which is demonstrated by phylum wise composition of bacteria in Figure 1A (Yong et al., 2017; Tokuda et al., 2018; Bozorov et al., 2019; Gawande et al., 2019; Shah et al., 2020; Asimakis et al., 2021; Deguenon et al., 2021; He et al., 2021; Tegtmeier et al., 2021; Yu et al., 2021; Csorba et al., 2022; Li S. et al., 2022; Majumder et al., 2022; Wang et al., 2022). We have noticed with taken examples that Proteobacteria, Firmicutes and Actinobacteria were the most dominant phyla present in the insect gut could be due to their diverse roles in nutrition. In our studied examples it is found that overall, the most abundant bacterial taxa at the phylum level were Proteobacteria except in termites where Spirochaetes are abundant. Phylum Firmicutes secured second position in bacterial composition in insect. Actinobacteria showed less symbiotic association in insect gut Figure 1B.

**Figure 2:**
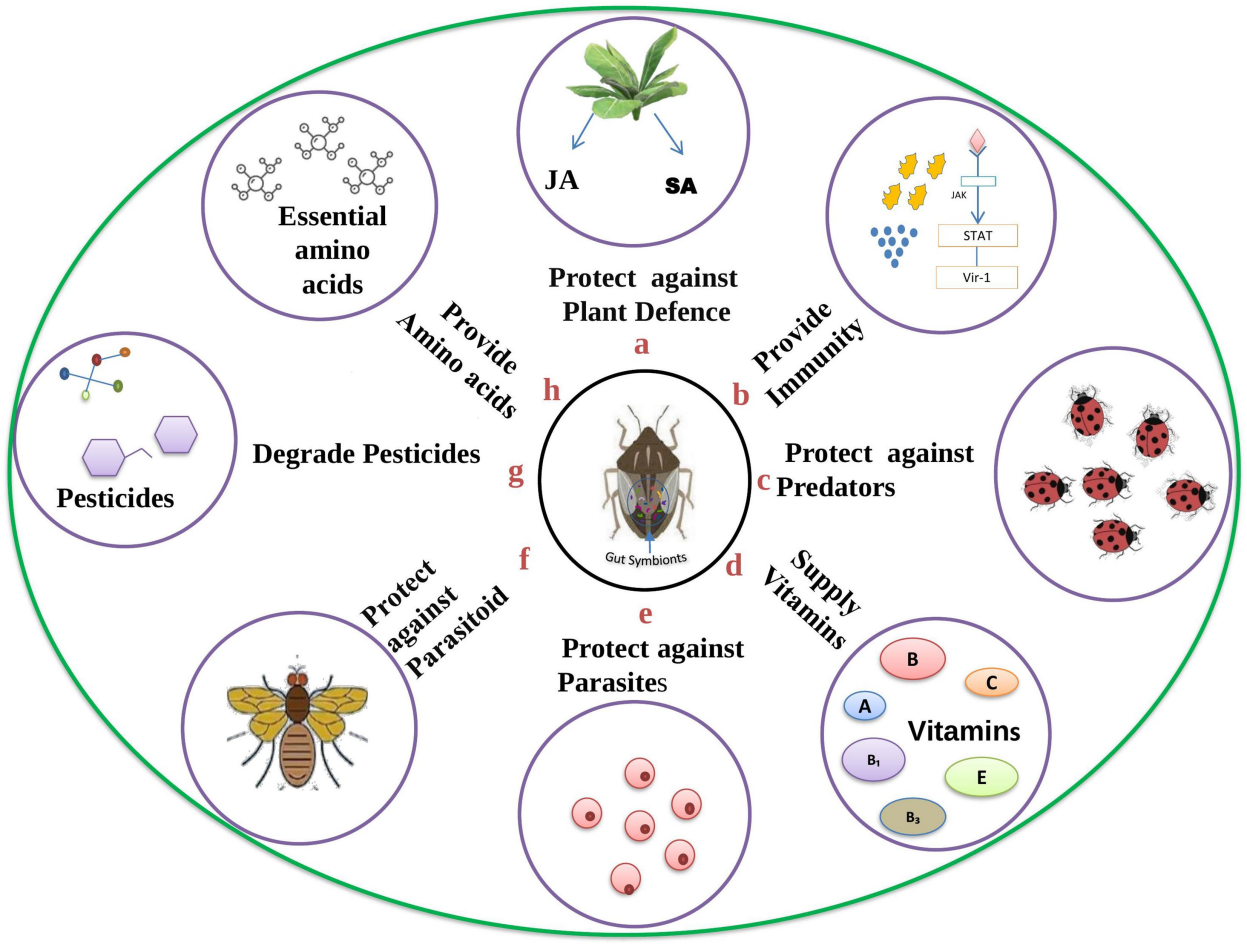
Diverse roles played by gut symbionts in the insect host body: The given figure depicts the various roles played by gut symbionts in the insect host body. **(A)** Plants secrete jasmonic acid (JA) and salicylic acid (SA) as a defence mechanisms against insect pests, but gut symbionts can degrade and protect the insect from these plant defences; **(B)** symbionts play a crucial role in shaping insect immunity *via* various pathways; **(C)** insects can change their morphological features due to the presence of symbionts which in turn helps the host in getting protection from predators; **(D)** insects feed on plant sap which is devoid of essential nutrients, but the presence of symbionts satisfies the need for essential nutrients; **(E)** symbionts secrete counter effective molecules that act on pathogens and prevent insects from becoming infected; **(F)** parasitoid wasp lays eggs on insect bodies, but once the egg hatches into a larva, the presence of symbionts halt the further development of the larva and the host is protected; **(G)** gut symbionts can break down pesticides into less harmful products; **(H)** insects cannot synthesize essential amino acids, but symbionts synthesize and fulfill the need of the host insect.

Insects can adapt to different ecological niches through symbiotic microbes in their digestive tract. Microbial mutualism is advantageous to the insect in many ways, e.g., by interaction with host plants, pathogens, parasitoids, parasites, or predators. A number of enzymes found in gut symbionts, which are located in the specialized cells, offer dynamic support to the host insects (Blow and Douglas, 2019). Studies have also proven that the microbial symbiotic association in the digestive tract has affected abiotic interactors such as temperature and the influence of inorganic toxins in the host system. The symbionts can alter their host viability by implementing traits essential to survival over a wide range of temperatures. In this way, most of the gut symbionts dominate the abiotic niche space, facilitating various adaptations in the host to cope with new habitats and changing environments (Lemoine et al., 2020). Insects with restricted diets usually show close symbioses with bacteria. These symbiotic relationships are often employed to meet nutritional needs (Buchner, 1965; Blow and Douglas, 2019). Most often, these symbiotic microorganisms supply enzymes to get rid of detrimental phytochemicals or help break down difficult polymers. They also supplement the host’s limited diet with vitamins, essential amino acids, and other nutrients (Douglas, 1998; Douglas, 2009; Douglas, 2015). Various essential roles of gut symbionts are portrayed in Figure 2.

### 2.1. Role of symbionts in nutrition

Insects are cosmopolitan and can feed on diverse diets, consequently adapting to extreme environments (Ishikawa, 2003). Usually the foods consumed by insects are deficient in one or another of the nutrients supplemented by their gut symbionts (Tamas et al., 2002). Studies on insect evolution have highlighted the importance of gut symbionts in increasing insect survival rates. They do this by helping insects meet their nutritional needs, providing them with nutrients directly, or introducing new metabolic pathways that allow insects to live in different environments (Wernegreen, 2004).

**Figure 3:**
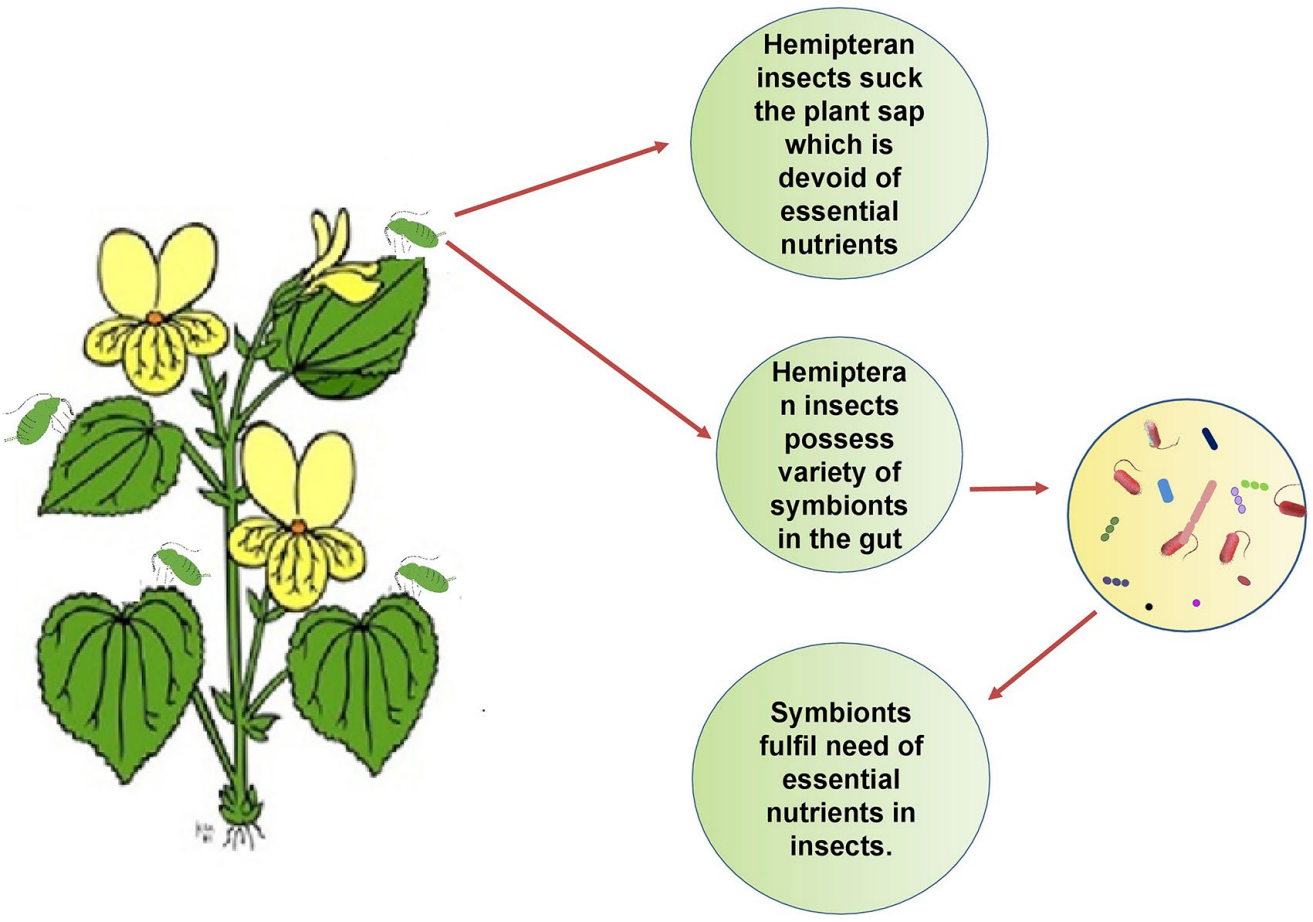
Symbionts help in the provision of essential nutrients: Plant sap does not provide the complete ingredients of the diet, so hemipteran insects are dependent on gut symbionts to get essential nutrients.

**Figure 4:**
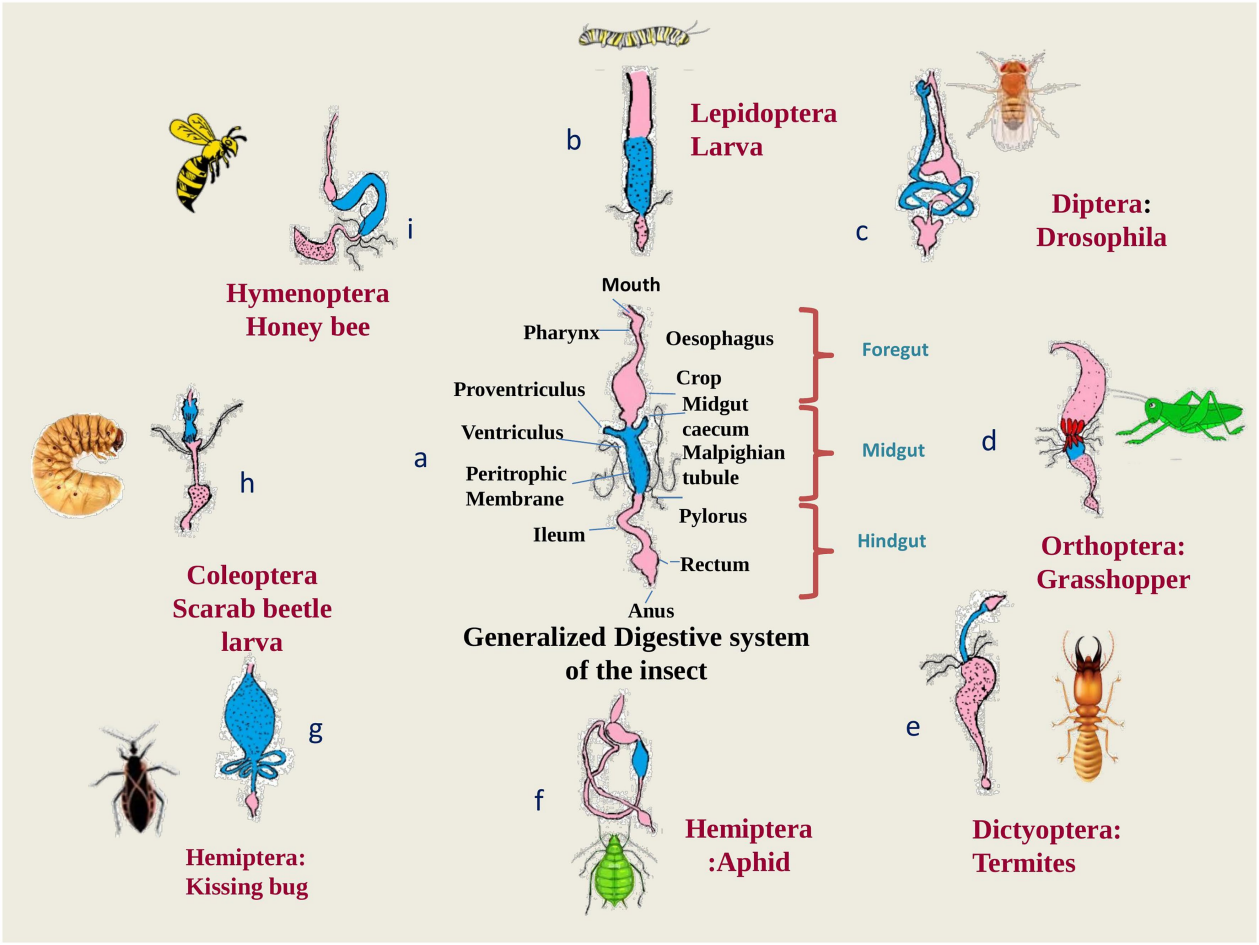
Various modifications in the gut structure among the insect orders to accommodate gut symbionts: **(A)** generalized digestive system of insects with a peritrophic matrix in the midgut (dotted lines). Pink regions indicate the foregut and hindgut, blue regions indicate the midgut. Black dots represented colonies of symbionts; **(B)** digestive system (D.S.) of a caterpillar of moth showing the presence of symbionts in the region of midgut as well as hindgut; **(C)** D.S. of Drosophila shows the presence of symbionts in the midgut region only; **(D)** D.S. of Grasshopper showing the distribution of symbiont in the whole digestive system; **(E)** D.S. of termites showing symbionts mainly in the hindgut; **(F)** D.S. of aphid showing the presence of symbionts along the internal lining of hindgut; **(G)** D.S. of Kissing bug which shows enlarged midgut to store packets of symbionts; **(H)** D.S. of Scarab beetle larva which shows the presence of symbionts in the hindgut; **(I)** D.S. of honey bee showing the presence of symbionts in hindgut region (Buchner, 1965; Chapman, 1998; Fukatsu and Hosokawa, 2002; Brune, 2010).

A diverse fauna of symbionts and their effectiveness in nutrition has been investigated in several crop pests, notably in whiteflies, aphids, treehoppers, sharpshooters, bugs and psyllids, i.e., hemipteran sap-sucking insects (Buchner, 1965; Douglas, 1998; Thao et al., 2000; Thao and Baumann, 2004; Luan et al., 2015). High throughput genome sequencing has backed the fact that the genes which are involved in symbiosis pathways, are highly conserved in insects with an improper diet that lacks nutritional components (Buchner, 1965; Moran et al., 2005a). Furthermore, the insects that harbor the bacterial symbionts feed exclusively on plant sap from phloem and xylem, which are rich in sugars but poor in vitamins and amino acids (Redak et al., 2004). The Figure 3 shows how gut symbionts help insects to get the food they need to survive. As already mentioned, physiological conditions in gut are the most favorable for symbionts acquisition yet the colonization of symbionts in the various parts of digestive tract are the peculiar feature for respective insect order (Eichler and Schaub, 2002) as depicted in Figure 4. The midgut epithelium of larva of olive fly is lined by symbiont *Candidatus Erwinia dadicola*.

The *Candidatus Erwinia dadicola* has an outstanding potential to utilize urea and non-essential amino acids as a source of nitrogen for adult and developing insects (Estes et al., 2009; Ben-Yosef et al., 2014).

The glassy-wing sharpshooter, *Homalodisca vitripennis,* has been shown to harbor a symbiotic pair of *Sulcia* and *Baumannia cicadellinicola* in the bacteriome of midgut. The genome studies of *Sulcia* revealed that it can encode entire metabolic pathways for eight essential amino acids such as arginine, tryptophan, phenylalanine, lysine, isoleucine, threonine, valine, and leucine. On the other hand, the remaining two essential amino acids; Histidine and methionine, along with many vitamins and cofactors, are encoded by the genome of *Baumannia cicadellinicola,* the other member of the symbiotic pair. The pair of this symbiotic microflora meets the need for essential amino acids and vitamin cofactors in the *Homalodisca vitripennis* (Moran et al., 2005a; McCutcheon and Moran, 2007).

Members of the genera *Buchnera* and *Wigglesworthia* are persistent obligate nutritional symbionts of sapsucker aphids and tsetse flies. Another great example of such an obligatory symbiotic relationship is *Ishikawaella capsulata* and the plataspid stinkbug *Megacopta punctatissima. Ishikawaella capsulata* shares striking similarities with *Buchnera.* This helps the host to gain the benefit of essential amino acids encoded by a highly reduced genome. Although *I. capsulata* and *Buchnera* share the symbiotic functions, they are located at different locations within the hosts. *Buchnera* is an endosymbiont, whereas *Ishikawaella* resides in the extracellular tissue of the posterior midgut (enlarged portion) of the adult stinkbug (Hosokawa et al., 2006; Nikoh et al., 2011). Table 1 enlists diverse symbionts helpful in digestion.

**Table 1:** List of symbionts helpful in digestion.

Bacterial symbiont	Host insect	Symbiont localization	Nutrient supplemented	Reference
*Carsonella* sp.	Psyllids	Bacteriocytes	Amino acids	Thao et al. (2000)
*Buchner* asp.	*Acyrthosiphon pisum* *Schizaphis graminum* *Baizongia pistacea*	Bacteriocytes	Essential amino acids	Douglas et al. (2006) Tamas et al. (2002) van Ham et al. (2003)
*Trembalya* sp.	Mealy bug	Bacteriome	Amino acids	Baumann (2005)
*Wigglesworthia*	*Glossina* spp.	Bacteriocytes	Vitamin B complex	Zientz et al. (2004)
*Baumannia*	*Homalodisca coagulate*	Bacteriocytes	Amino acids	Moran et al. (2003)
*SOPE (Sitophilus oryzea)*	*Sitophilus oryzea*	Bacteriocytes	Vitamins	Heddi et al. (1998)
*Sodalis*	*Glossina* spp.	Gut and numerous tissue	Nutrition	Aksoy (1995)
*Nocardia*	*Rhodillus* spp.	Midgut	Vitamin B complex	Eichler and Schaub (2002)
*Blochmannia*	*Camponotus* spp.	Gut	Amino acids and fatty acids	Gil et al. (2003)
*Gilliamella apicola*	*Honey Bee*	Gut	Pectin Degradation	Engel et al. (2012)
*R. prolixus*	*R. rhodnii,*	Gut	Vitamin B	Eichler and Schaub (2002)
*Ishikawaella capsulatus*	Plataspid stinkbugs	Gut	Essential amino acids	Nikoh et al. (2011)

### 2.2. Symbionts: Central regulators of signaling pathways

As discussed earlier, the digestive tract of most insects harbors a complex community of symbionts, and many of them mediate homeostasis with epithelial cells of gut lining. The presence of symbionts modulates the variety of physiological along with nutrition, anti-plant defence, and protection from pesticides. Changes in diet, physiological activities and environmental conditions f host insects can affect symbiont load and further homeostatic relationship between symbionts and epithelial tissue (Clark et al., 2015). Commensals always try to evade the host insect’s immune system while gut epithelium has the control over microorganisms. Nonetheless, the gut epithelium must elicit a subtle and timely response of in order to receive signals of fluctuations in the microbial population. The molecular mechanism underlying this immune response is elucidated by Xiao et al. (2017) in *Aedes aegypti* and *Drosophila melanogaster.* Expression of Dual oxidase is controlled by Mesh protein located at the gut membrane (Huang et al., 2015). Duox plays a crucial role in controlling the bacterial population in the insect gut. Duox controls bacterial proliferation *via* the arrestin-mediated MAPK JNK/ERK phosphorylation cascade, which forms the basis of insect gut homeostatic physiology (Xiao et al., 2017).

Furthermore, the development of the digestive system of insects is under the control of intestinal bacteria since this symbiotic association with bacteria can lead to reestablishment of epidermal cells and induction of intestinal stem cells proliferation. Gamma-proteobacteria from honey bee (*Apis mellifera*) codes for pectin-degrading enzyme useful in breakdown of pollen walls (Engel et al., 2012). These gut symbionts facilitate variety of roles including combat against environmental changes, triggering individual weight gain *via* vitellogenin synthesis and insulin signaling. Symbiotic bacteria can modulate intestinal pH and its redox capacity by synthesizing short chain fatty acids that induce changes in insulin/insulin-like growth factor signaling. Two ILP genes and two putative insulin receptors are found in honeybees. Furthermore, vitellogenin synthesis in honey bees is directly linked to insulin/insulin-like growth factor signaling. A significant increase was observed in the expression levels of *ilp1, ilp2, inR1* and *vg* genes in normal bees compared to sterile bees (Zheng et al., 2017).

Similarly, the enzymes produced by gut symbionts have a significant role in growth and development of the host insect by producing enzymes as has been obseved in *Drosophila*. In the gut of fruitfly the resident bacterium *Acetobacter pomorum* produces pyrroloquinoline quinone-dependent alcohol dehydrogenase (PQQ-ADH) which modulates homeostasis by controlling intestinal stem cell activity, growth, body size and energy utilization. Similarly, the symbiotic bacteria *Lactobacillus plantarum* can cause changes in sex pheromone levels when receiving different diets. Diet dependent mating biases are observed in *Drosophila* (Sharon et al., 2011). In addition, gut symbionts such as *Acetobacter*, *Lactobacilli*, and *Enterococci*, of *Drosophila* are recognized as central regulator that controls host physiology and behavior. In the similar studies it is found that microbiome derived signals are responsible for aggression in both male and female *Drosophila*. The aggressive *Drosophila* has shown higher levels of tyrosine decarboxylase 2 (Tdc2) and tyramine beta-hydroxylase (enzymes required for synthesis of octopamine). In this way *Drosophila* promote aggressive behavior *via* higher levels of octopamine (Jia et al., 2021). Similarly, development of this fruit fly is also symbiont dependent. The expression of imaginal morphogenesis protein-Late 2 (Imp-L2), depends on bacteriocyte harboring *Acetobacter* and *Lactobacilli* (Lee et al., 2022). Studies have shown that the symbionts harboring the gut can activate gut nerves to send signals to brain nerves (Forsythe and Kunze, 2013). Along with this neural activity, symbionts have control over host activities. In the gut of locust, the symbiont *Pantoea agglomerans*, synthesize cohesion pheromone required for gathering of locusts (Dillon et al., 2002).

### 2.3. Role of symbionts in fighting plant defence systems

Insects and plants have evolved simultaneously, and both are cohabiting together for more than 350 million years. Insects and plants try to evolve and acquire unique traits of defence against each other for their successful survival. Attack by insects has led to the initiation of defence system in plants to recognize signals from damaged cells and foreign molecules, which provoke immune response in plants against the attacking insects. Morphological structures such as thorns, hair, trichomes, spines and the secretion of secondary metabolites such as phenol, alkaloids, terpenoids etc. have been acquired by plants, which in turn cease the development of the insect pests (Dudareva et al., 2006; Howe and Jander, 2008; Arimura et al., 2009; Rani et al., 2009; Verhage et al., 2010; Hare, 2011; War et al., 2011).

The defensive strategy utilizing secondary metabolites is known as constitutive defence. This defence involves the secretion of various volatile secondary metabolites. In addition to the constitutive defence, plants acquire induced defence carried out by phytohormones such as salicylic acid (SA) and jasmonic acid (JA) in response to the attack by herbivore insects. The JA pathway gets triggered upon attack by insects with chewing-biting mouth parts whereas the SA pathway gets promoted by the attack of piercing-sucking herbivores (Glazebrook, 2005; Schoonhoven et al., 2005; Kawazu et al., 2012).

Most insect pests feed on plant phloem as a source of nutrition. In response, the plant has its mechanism to invite natural enemies of the insect pest or secrete toxic chemicals to get rid of the latter (Casteel et al., 2012; Su et al., 2015). To overcome this plant defence mechanism, insect pest gut symbionts play a crucial role by conferring various defence strategies against these natural enemies and toxic secretions of the host plants (Frago et al., 2012; Oliver et al., 2014). These strategies facilitate the insect host to produce counter-effector molecules against toxic chemicals secreted by plants and restrict the development of enemy animals by limiting their egg deposition capacity (Bruessow et al., 2010) and oral secretions (Consales et al., 2012; Chung et al., 2013).

Studies have speculated that mutualistic symbiont associations in insects carry mediation in direct and indirect interactions between insects and their host plants *via* many strategies (Su et al., 2013). Aphids being an economically important pest, damage crops by disturbing phloem component which results into activation of SA and JA defence pathways in various crops, e.g., Sorghum, *Arabidopsis*, Tomato, Cabbage, Soybean and Potato. To overcome these plant defence patterns, symbionts from the insect gut interfere in the signal transduction pathway by suppressing the expression of defence pathway genes resulting in altered primary and secondary metabolites (Body et al., 2013; Giron et al., 2013; Zhu et al., 2014; Sugio et al., 2015). In addition, symbionts can introduce novel metabolic pathways to detoxify secondary metabolites of plants in the host insects (Moran et al., 2008; Douglas and Dobson, 2013). All these studies estimate that symbionts may have a potential role in manipulating insect and plant interactions. Table 2 depicts various examples of suppression of plant pathways by insect symbionts.

**Table 2:** List of symbionts acting against plant defence.

Insect host	Symbiont	Plant pathway	Reference
Wheat aphid *Sitobion miscanthi*	*Hamiltonella defensa*	ceases the expression of genes related to SA and JA defence pathways	Li et al. (2019)
Coloradopotato beetle, *Leptinotarsa decemlineata*	Bacteria from salivary secretions	Invokes the SA-related plant defence pathway and suppresses JA pathway	Chung et al. (2013)
*Bemisia tabaci*	*Hamiltonella defensa*	Suppression of the JA pathway in a tomato plant	Su et al. (2015)
Corn root borer *Diabrotica virgifera*	*Wolbachia*	Suppression of plant defence	Barr et al., 2010
Tomato psyllid, *Bactericerca cockerelli*	*Candidatus* spp.	influences the expression of defence-related genes	Casteel et al. (2012)

**Figure 5:**
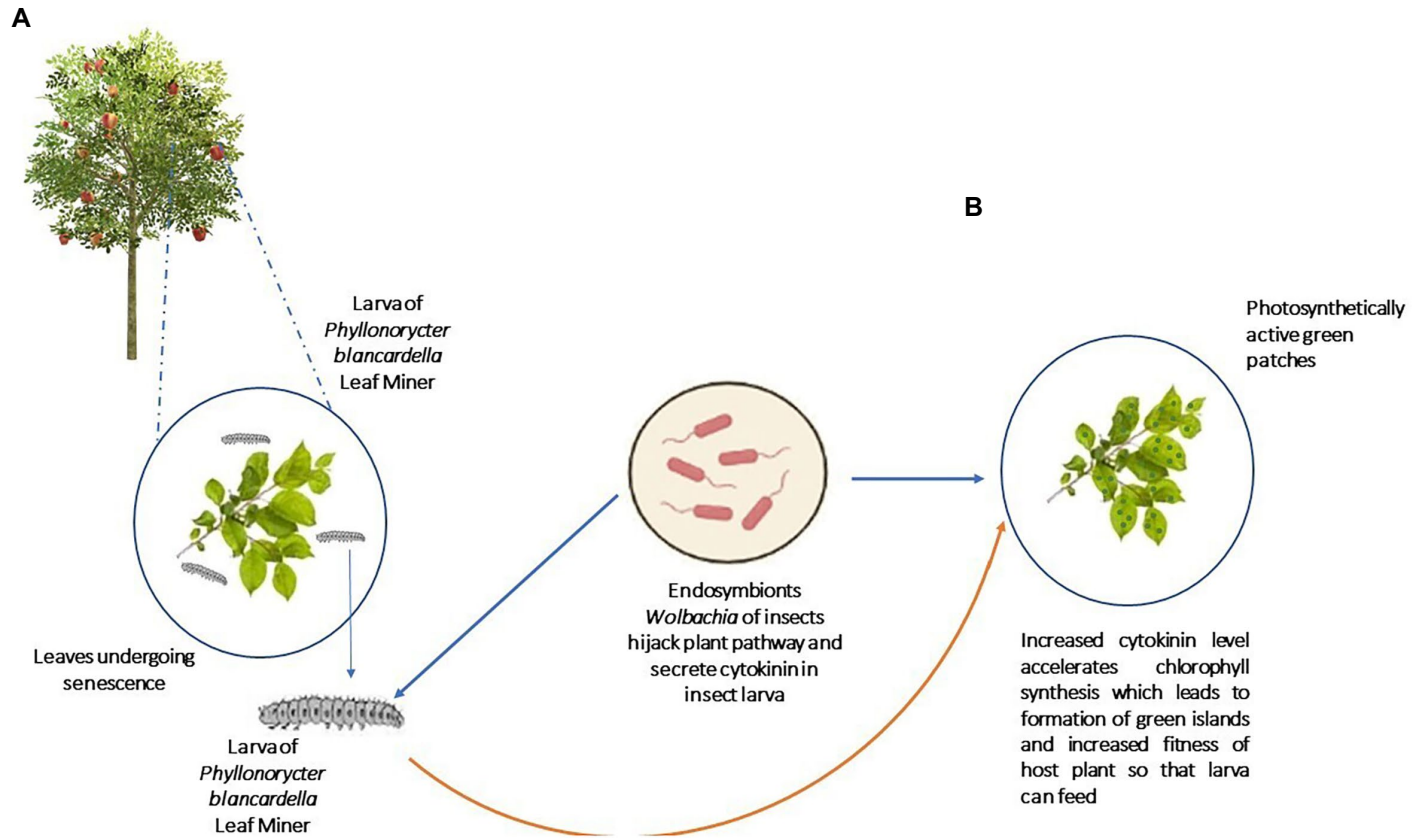
Hijack of plant pathway by symbionts: Gut symbionts of leaf miner *Phyllonorycter blancardella* can mimic the plant pathways where it secretes cytokinins and fools the plant resulting in chlorophyll production. **(A)** Before infestation by *Phyllonorycter blancardella*, **(B)** After infestation by *Phyllonorycter blancardella* (Production of green islands of chlorophylls as patches; Kaiser et al., 2010; Body et al., 2013).

Besides modulating SA and JA pathways, insect symbionts also hijack other crucial pathways of plants that benefit the host insects (Panteleev et al., 2007). As depicted in Figure 5, the experimental removal of *Wolbachia* bacteria from its host, *Phyllonorycter blancardella,* apple tree leaf miner resulted in the loss of cytokine-induced green islands on apple tree leaves and reduced cytokinin levels in the larva as well (Kaiser et al., 2010; Body et al., 2013). These results show that the symbionts can modulate the phytohormonal profile of mined leaves and deliver cytokinins, which are synthesized in the insect body (Kaiser et al., 2010; Body et al., 2013). Cytokinins inhibit senescence, maintain chlorophyll content, and control the nutrient flow in plants (Gan and Amasino, 1995; Walters et al., 2008).

Instead of modulation, in some cases, insect gut symbionts are thought to counteract or inhibit plant defences as observed in cigarette beetles *Lasioderma serricorne* (Dowd and Shen, 1990). The symbionts of these insects produce enzymes that neutralize plant defence compounds. Plant defence product terpenoids get degraded by enzymes produced by the microbial symbionts of the mountain pine beetle, *Dendroctonus ponderosae* (Boone et al., 2013). Another pathway of metabolite degradation is observed in the bacteria cohabiting in the gut of *Dendroctonus ponderosae*. These bacteria are enriched with genes responsible for terpene degradation and hence can degrade monoterpene and diterpene acids, which are toxic to beetles (Adams et al., 2013). Furthermore, one of the plant’s secondary metabolites, oxalate is broken down by oxalate decarboxylase encoded by the *ode* gene of the plasmid of *Ca. I. capsulata* suppressing plant defence (Franceschi and Nakata, 2005; Hosokawa et al., 2006; Nikoh et al., 2011).

### 2.4. Role of symbionts in protection against external biotic threats

#### 2.4.1. Protection against parasitoid

Besides nutritional and defence benefits, symbionts are also known to play crucial roles in shaping ecological interactions between the natural enemies and the insects. Most insects possess an endogenous system of gut microflora to gain protection against natural enemies like parasitoids. Moreover, studies have shown that symbionts can modify insect fecundity rate as well as modulate interactions of insects with natural enemies or plant pathogens (Engelstädter and Hurst, 2009; Ferrari and Vavre, 2011; Frago et al., 2012; Biere and Bennett, 2013; Sugio et al., 2015; Chuche et al., 2016). Symbionts of insects have played a pivotal role in protecting insects from parasitoids. Parasitoids lay their eggs outside or inside of the insect’s body, mostly affecting the larval stage. Damage was caused by the piercing activity of parasitoid larvae, most commonly hymenopteran wasps.

Aphids are thought to be benefitted by their facultative bacterial endosymbionts through increased resistance to parasitoids. Facultative symbionts of aphids regulate the susceptibility of the latter to parasitism or predation (Xu et al., 2009). Peach-potato aphid, *Myzus persicae*, is often attacked by hymenopteran parasitoids. The role of *Hamiltonella defensa* and *Serratia symbiotica,* symbionts of these aphids in defending against the parasitoid wasp *Aphidius colemani* has been well documented*. Hamiltonella defensa* increases the life span of aphids by interfering with oogenesis in a parasitoid wasp. These toxic effects are produced by bacteriophage APSE of the symbiont. In another study *Myzus persicae* was found to get protection from parasitoid *Aphidius colemani,* due to the presence of facultative endosymbiont, *Regiella insecticola* (Oliver et al., 2003, 2005; Moran et al., 2005b; Vorburger et al., 2009; Schmid et al., 2012; Brandt et al., 2017). These studies suggest that many species of endosymbiotic bacteria may protect their hosts from their natural enemies.

The mutualistic association between a pea aphid and its symbionts offers insights into the strategies implemented by symbionts to protect the host from parasitoids. Huge variations in the clones of pea aphids (*Acyrthosiphon pisum*) are observed *via* the bacterial symbiont *Hamiltonella defensa.* When attacked by biological pest control agent parasitoid *Aphidius ervi,* symbiont *H. defensa* reduces the release of volatile compounds by aphid-infested plants so that wasps cannot detect the aphids and cannot visit the plant (Frago et al., 2012, 2017). Similar studies have shown that in the *Acyrthosiphon pisum* symbionts; *Hamiltonella defensa* and *Serratia symbiotica* confer protection against parasitoids *Aphidius ervi* and *Aphidius eadyi* by restricting the development of larval stages (Oliver et al., 2003; Ferrari et al., 2004; Oliver et al., 2005). When the feasibility of the *Acyrthosiphon pisum* for the parasitoid *Aphidius ervi* is checked by artificially infecting the aphid with the primary symbiont *Buchnera aphidicola* and a vertically transmitted secondary symbiont, it is observed that the infection of the symbiont results in high mortality of developing parasitoid larvae. Thus, symbionts appear to provide resistance against parasitoid attacks (Oliver et al., 2005).

**Figure 6:**
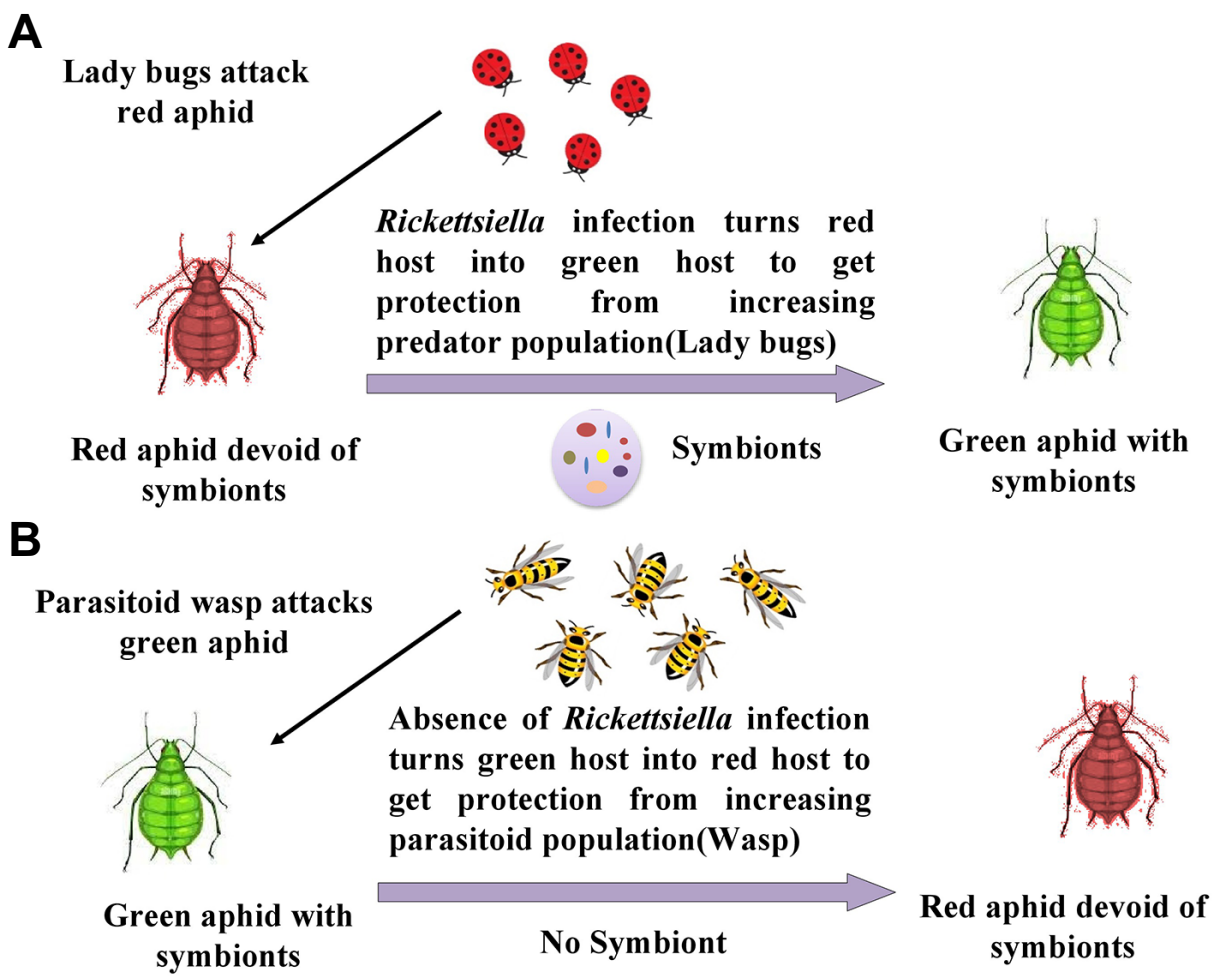
Change in body color in response to acquisition by Rickettsiella: **(A)** When red aphids are attacked by predators like ladybird beetle, there is the accumulation of gut symbionts *Rickettsiella,* which turn red aphids into green aphids; **(B)** When green aphids are attacked by parasitoid like wasps, the population of *Rickettsiella* get reduced turning green aphids into red aphids (Libbrecht et al., 2007; Tsuchida et al., 2014).

*Acyrthosiphon pisum* acquires benefits from *Rickettisella* infection, which protects the insect from the attack of parasitoids as well as predators. Usually, this aphid is present in nature in red color, and predators such as ladybird beetles mainly feed on red-colored pea aphids. As the population of ladybird beetles increases in the field, the *Rickettisella* infection of the aphid leads to increased blue–green quinone pigment synthesis in the host. This mechanism imparts the green color to the host, ultimately amid green crops; green aphids get camouflaged and protected from ladybird beetles. On the other hand, parasitoids such as wasps attack green-colored aphids. Whenever there is an attack by parasitoids, these symbionts turn the green host into the red host, and aphids get protected from parasitoids (Libbrecht et al., 2007; Tsuchida et al., 2014). This mechanism is illustrated in Figure 6.

#### 2.4.2. Protection against predators

Some of the mutualistic symbionts aid their host insects in tackling predators. These symbionts can encode compounds that are toxic to predators. It is observed that the *Paederus* beetle gains protection from wolf spiders through their endosymbionts. These symbionts produce the polyketide toxin “Pederin,” which is harmful to wolf spiders (Kellner, 1999; Kellner, 2001; Kellner, 2002; Piel, 2002; Piel et al., 2004). Sometimes these symbionts compete with natural enemies for limited resources such as lipids and cholesterol. Competition for resources results in the elimination of predators. In addition to these defence systems, microbes can synthesize a bundle of harmful toxins which can retard predator invasion (Caragata et al., 2013; Paredes et al., 2016).

#### 2.4.3. Protection against pathogens

Like other organisms, insects are also susceptible to a wide range of microbial pathogens such as protozoans, viruses, fungi, and bacteria. To avoid pathogens, insects exhibit either escape or avoidance behavioral patterns. Insects also possess other defence mechanisms such as physical barriers, and cellular and humoral responses involving the production of antimicrobial peptides as a mode of protection against pathogens (Lemaitre and Hoffmann, 2007; Sheehan et al., 2018). The integral symbiotic microbial association supports or facilitates the various defence mechanisms of insects. Studies have shown that the artificial transfer of *Wolbachia* from Drosophila to mosquito results in a boosted innate system against human pathogens such as Zika virus, West Nile virus, Chikungunya, Dengue, and *Plasmodium* (Caragata et al., 2019). The microbial symbionts can also synthesize a bundle of harmful toxins which affect the physiology of microbial pathogens by destroying the cellular components, modifying signaling pathways, and metabolic synthesis in pathogens (Caragata et al., 2013; Paredes et al., 2016). Another striking example of microbe-mediated protection against protozoan infection is found in the cardia of *the Anopheles gambiae* gut (Warr et al., 2007). The gut microbes of mosquitoes help to invoke an immune response against *Plasmodium via* the secretion of several anti-plasmodium factors and affect parasite development in the mosquito gut (Dimopoulos et al., 2007).

The protection against fungal pathogens in insects is observed in a solitary hunting wasp., *Philanthus triangulum,* the European beewolf. The female of these wasp harbors *Streptomyces* bacteria in specialized antennal glands, and just before oviposition, these bacteria are transferred to the brood cells. After emerging from the eggs, the larva acquires the bacteria deposited in the brood cells, which are later on transferred onto the walls of the cocoon. These bacteria produce specific antibiotics which protect the larva from fungal pathogens and enhance the survival rate of the insect. The presence of *Regiella insecticola,* the facultative symbiont in aphids limits the growth of *Pandora neoaphidis,* the fungal pathogen (Ferrari et al., 2004).

### 2.5. Role of symbionts in immunity boosting

The midgut of insects is furnished with a heterogeneous defence system. The presence of gut microflora is the basis for the existence of dynamic immune system. Another means of defence includes the preparation of a protective matrix. Most insect midguts can secrete a chitin-based peritrophic matrix where chitin microfibrils are embedded in a protein-carbohydrate matrix (Terra, 1990). This peritrophic matrix is selectively permeable and recognizes the nutrients, defensins, and digestive enzymes but protects the epithelium from a direct encounter with microorganisms or toxic molecules. This physiological barrier has the power to lower the negative impact of bacterial load (Daffre et al., 1994; Hultmark, 1996; Dubreuil et al., 2001).

The gut epithelium of honey bee (*Apis mellifera*) harbors colonies of *Snodgrassella alvi* and *Gilliamella apicola* in the form of a dense biofilm that inhibits the entry of pathogens (Kwong and Moran, 2013). Disturbance in these bacterial colonies from the gut may result in infection of opportunistic pathogens like *Serratia marcescens*, causing higher mortality rates in honeybees (Kwong and Moran, 2013). Gut symbionts are thought to enhance the immune system by modulating pH and oxygen levels, producing short-chain fatty acids which subsequently suppress the pathogen virulence in the bees (Pickard et al., 2017; Zheng et al., 2017). Along with this, these symbionts up-regulate the expression of compounds of the immune system such as apidaecin, which helps in pathogen clearance (Emery et al., 2017; Kwong et al., 2017).

### 2.6. Role of symbionts in pesticide degradation

The endosymbionts of insects are thought to offer protection against both natural enemies and man-made synthetic chemicals. According to long evolutionary associations, endosymbionts can keep their host alive in adverse conditions for their mutual benefit. The assembly of various chemical-degrading microbial enzymes entails the successful elimination of hazardous chemicals from the host body. Earlier research on the effectiveness of insecticide resistance mainly focused on factors like target site insensitivity, proliferated metabolic detoxification, and inability to cross the epidermis. However, symbiont-mediated detoxification is becoming an emerging trend in insect pest management (Siddiqui et al., 2022).

For the rapid removal of pests, the application of pesticides is the most common method used worldwide. Pesticides of the organophosphate group are applied extensively, as they show oral and percutaneous arthropod-specific toxicities like inhibiting the activity of acetylcholine esterase causing neurotoxic effects in the insects (Stenersen, 2004). To survive, the pests usually develop insecticide resistance in response to extensive use of pesticides. This has become the main obstacle in pest control management these days (Hemingway and Ranson, 2000; Hemingway et al., 2002; Després et al., 2007; Heckel, 2012; Alyokhin and Chen, 2017).

There were hardly any reports of pests developing insecticide resistance till 1980. However, one study hinted at the potential mechanism of detoxification of insecticides in the apple maggot *Rhagoletis pomonella* that causes significant damage to apple plants. To overcome and tackle the infection and the subsequent crop loss, organophosphate was applied in enormous amounts. In these apple maggots, Boush and Matsumura detected a pathway related to the detoxification of organophosphate, exhibited by a bacterial symbiont of the apple maggot (Boush and Matsumura, 1967). In addition to this, the idea of symbiont bacteria-mediated degradation of insecticides is supported by the study of the midgut symbiont *Burkholderia* of the bean bug *Riptortus pedestris* (Kikuchi et al., 2011; Kim and Lee, 2017). *Burkholderia* can degrade an organophosphate pesticide, fenitrothion, conferring insecticide resistance to the host bean bug. Similarly, *Cavelerius saccharivous*, another bug, showed the presence of pesticide-degrading *Burkholderia* symbionts when collected from fields that were regularly sprayed with fenitrothion (Kikuchi et al., 2012).

A leafhopper bug, *Riptortus pedestris*, possesses a specific *Arsenophonus* strain that has metabolic capabilities to detoxify pesticides (Pang et al., 2018). Likewise, the fly *Bactrocera dorsalis* (Diptera: Tephritidae) detoxifies trichlorfon using a phosphatase hydrolase enzyme, produced by its gut symbionts, *Citrobacter* sp. The host insects *Blatella germanica, Plutella xylostella, Lasioderma serricorne, Anopheles stephensi, Nilaparvata lugens, Bactrocera dorsalis, Culex pipiens, Spodoptera frugiperda, and Callosobruchus maculatus* are known to acquire and harbor specific gut symbionts in response to pesticide application (Shen and Dowd, 1991; Berticat et al., 2002; Almeida et al., 2017; Cheng et al., 2017; Soltani et al., 2017; Pietri et al., 2018; Xia et al., 2018; Akami et al., 2019a).

Kikuchi et al. (2011) have reported the presence of *Burkholderia* bacteria in stink bugs and Oriental chinch bugs (*Cavelerius saccharivorus*) from bean and sugarcane fields, respectively. *Burkholderia* is thought to degrade fenitrothion, imparting pesticide resistance to the insects. The stink bug uses a well-defined symbiont sorting mechanism to choose stink bug-associated beneficial bacteria (SBE-) or plant-associated beneficial bacteria (PBE-) *Burkholderia* (Kikuchi et al., 2011; Olivier-Espejel et al., 2011; Boucias et al., 2012; Garcia et al., 2014; Itoh et al., 2014a; Takeshita et al., 2015). A similar mechanism is found in the oriental fruit fly, *Bactrocera dorsalis* (Hendel), harboring the gut symbiont *Citrobacter freunde*, which is resistant to trichlorfon. Transfer of these bacteria takes place *via* vertical transmission while ovipositing and oral routes of the larva (Cheng et al., 2017). In addition to this, Almeida et al. (2017) showed the breakdown of pesticides by symbiotic bacteria; e.g., the fall armyworm larvae acquired resistance against chlorpyrifos, deltamethrin, lambda-cyhalothrin, spinosad, and lufenuron by harboring gut bacteria. *In vitro* studies on bacterial isolates exhibited strong esterase activity, which is crucial in the degradation pathway and utilizes carbon, nitrogen, and phosphorus for energy (Li et al., 2017). Similarly, bacteria like *Bacillus cereus* are shown to be capable of extracting carbon from the insecticide indoxacarb (Ramya et al., 2016). In addition, *Lactobacillus* from *in vitro* culture was also able to sequester organophosphates, causing reduced mortality in *Drosophila melanogaster,* when treated with insecticides (Trinder et al., 2016). Antibiotic treatment of *Spodoptera litura* (F.) symbionts resulted in reduced resistance to flubendiamide, indoxocarb, and chlorpyriphos (Gadad et al., 2016).

The reductive antibiotic approach, studied by various groups, highlights the presence and chemical degradation abilities of symbiotic microbes (Shen and Dowd, 1991; Almeida et al., 2017; Cheng et al., 2017; Soltani et al., 2017; Pietri et al., 2018; Xia et al., 2018; Akami et al., 2019b). Moreover, it has been discovered that this emerging insecticide resistance phenomenon is not confined to one insect class but can be found in varied insect groups having distinct ecological interactions and life histories. These findings provide insight into the interaction of chemical pesticides with insect symbionts. Most often, resistance mechanism machinery is encoded by the genomes of insects, but recent studies have discussed the role of specific gut microorganisms in the removal of toxic compounds (Hammer and Bowers, 2015; Itoh et al., 2018a). Heavy use of organophosphate compounds leads to disturbance in various ecosystems (Kikuchi et al., 2011). To overcome this situation, researchers are investigating pesticide degradation routes in the environment. Earlier studies also suggested the presence of MEP-degrading bacteria such as *Pseudomonas, Flavobacterium* (Kawahara et al., 2010), *Cupriavidus, Corynebacterium, Arthrobacter, Sphingomonas, Pandoraea, Dyella, Achromobacter, Ralstonia,* and *Burkholderia* in soil (Tago et al., 2006; Zhang et al., 2006; Kim et al., 2009; Itoh et al., 2014b). Burkholderia is thought to play a major role in metabolizing pesticides to satisfy the needs of carbon, nitrogen, and phosphorous. Genetic elements of this degradation pathway have been laterally transferred among diverse soil bacteria as well. These bacteria are acquired by insect pests too. For instance, *R. pedertris* acquires the symbiont from ambient soil in every host generation (Kikuchi et al., 2007).

**Figure 7:**
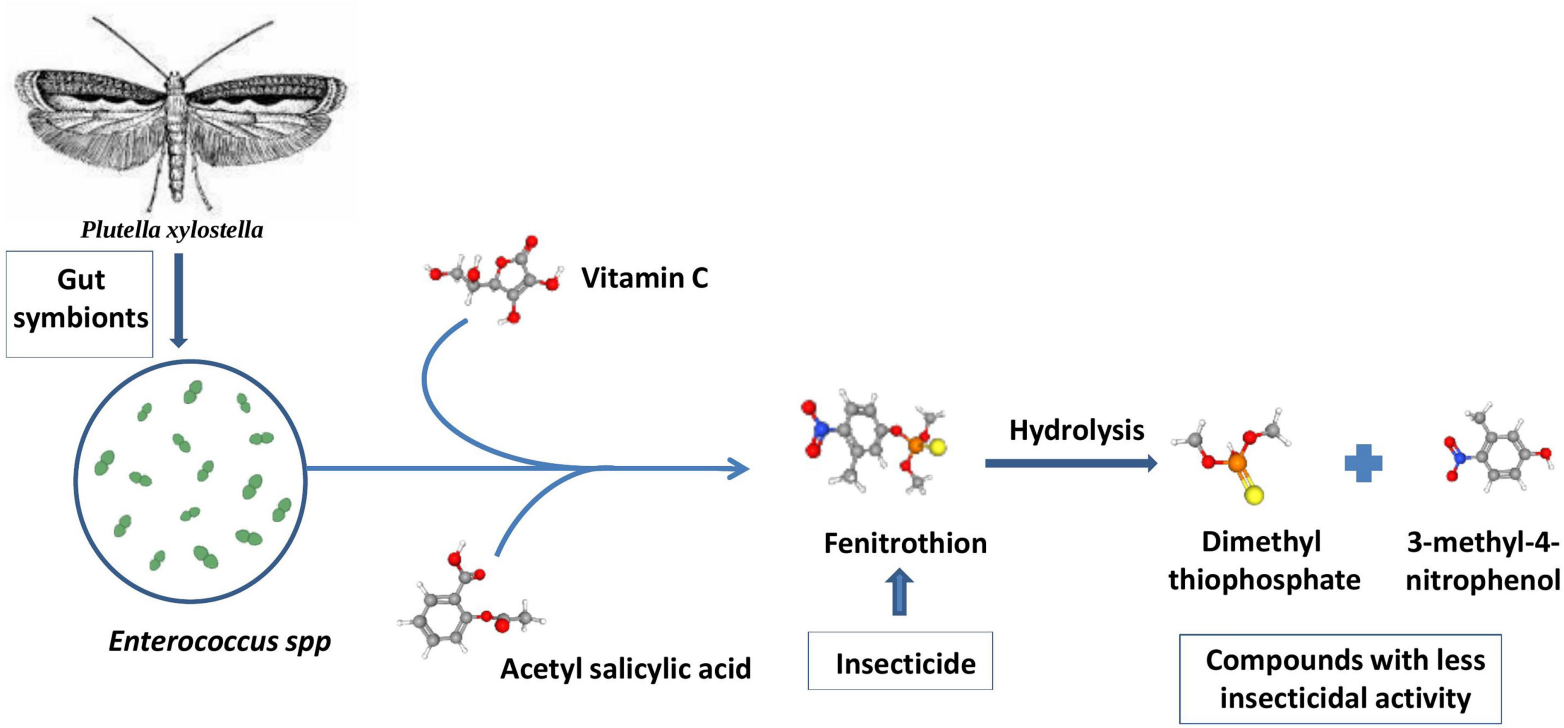
Pesticide degradation by gut symbionts in *Plutella xylostella*: The gut lining of *Plutella xylostella* is occupied with the gut endosymbiont *Enterococcus* spp. that degrades phosphate-based pesticides like fenitrothion into less harmful products such as 3-methyl-4-nitrophenol (Hayatsu et al., 2000; Xia et al., 2018).

All these studies suggest that repeated exposure to any toxic chemical may result in the evolution of the insect pest by acquiring appropriate symbionts that can degrade the pesticides effectively. The use of chemical pesticides has triggered the acquisition of pesticide-degrading bacteria. However, this resistance may be temporary and vanish in the next generation of the insect host (Kikuchi et al., 2012). This research paved the way to study symbiont-mediated pesticide detoxification and pesticide tolerance in insect pests. Subsequent studies related to the collaboration of microbes with insects to get rid of toxic chemicals and bio-transformation are thought to be complex phenomena (Pietri et al., 2018). Symbionts are thought to minimize the negative effects of insecticides *via* two mechanisms. One of the mechanisms involves the production of detoxification enzymes by the symbiont microbes upon exposure to insecticides. These enzymes catalyze the degradation of insecticides *via* co-metabolism or mineralization. In another mechanism, the immune system of the host insect interacts with gut symbionts and leads to cooperative insecticide resistance. For example, the attack of the pesticide chlorpyrifos on the immune system of the moth *Plutella xylostella* (Lepidoptera: Plutellidae) is prevented by combining the effect of the gut symbiont *Enterococcus* sp., vitamin C, and acetylsalicylic acid. These bacteria can hydrolyze fenitrothion into compounds like dimethyl thiophosphate and 3-methyl-4-nitrophenol that have little insecticidal activity. These bacteria can further metabolize the degradation product as a carbon source for their growth, as illustrated in Figure 7 (Hayatsu et al., 2000; Xia et al., 2018).

### 2.7. Some interesting case studies: The downfall of symbionts to increase insecticide susceptibility

Since the symbionts are harbored in special structures like crypts of the midgut or bacteriocytes in host insects, they are considered a “versatile organ” of insects (Sibao and Shuang, 2017). As mentioned earlier, symbionts play crucial roles for their hosts and have effects on nutritional requirements, overall development, reproductive functions, protection against parasites, predators, parasitoids, viruses, and fungal infections, detoxification metabolism, and control of host gene expression, etc. (Douglas, 1998; Stouthamer et al., 1999; Douglas et al., 2001; Oliver et al., 2003; Baumann, 2005; Montenegro et al., 2006; Douglas, 2009; Lee et al., 2017). Also, the symbionts have a very crucial role in the metabolic detoxification of plant allelochemicals and insecticides (Pavlidi et al., 2017; Itoh et al., 2018b). This detoxification is achieved either by breaking down the toxic chemicals or get rid of all toxins by controlling the expression of host genes (Kikuchi et al., 2012; Jones et al., 2015; Cheng et al., 2017).

The brown plant hopper, *Nilaparvata lugens*, is a notable pest of rice crops throughout East and Southeast Asia (Liu et al., 2005; Zhang et al., 2018). *Nilaparvata lugens* has developed resistance against several insecticides by increasing metabolic ability and overexpressing insecticide-degrading enzymes such as cytochrome P450 (P450) and glutathione S-transferase (GST), which play a vital role in detoxification (Vontas et al., 2002; Zhang et al., 2016). To demonstrate this pesticide resistance, a study was carried out where the *N. lugens* population was treated with antibiotics such as tetracycline and ciprofloxacin. The results showed that these antibiotics significantly collapsed the dominant population of symbionts such as *Wolbachia, Arsenophonus, Acinetobacter, Lactobacillus,* and *Klebsiella,* which resulted in increased insecticide susceptibility of the *N. lugens via* downregulating the expression of P450 and GST.

In *Drosophila* spp., gut bacteria are shown to stimulate the production of reactive oxygen species (ROS) in epithelial cells *via* enzymes. ROS stimulates the expression of NADPH oxidase 1 (NOX1) expression and affects the Nuclear factor E2-related factor 2 (Nrf2) pathway to regulate host detoxification metabolism (Jones et al., 2013). It is also observed that *Wolbachia* enhances pesticide detoxification gene CYP4CE1 expression in response to imidacloprid stress (Cai et al., 2021). Similar studies have shown that ciprofloxacin treatment led to the down-regulation of CYP6ER1, one of the P450 genes in *N. lugens*. This gene is thought to participate in the metabolism of varied insecticides, including sulfoxaflor, thiamethoxam, imidacloprid, and nitenpyram (Zhang et al., 2016; Jin et al., 2017; Mao et al., 2019).

## 3. Integrated pest management

The idea of IPM laid its foundation in the late 1950s. The concept is based on the ability of crops to tolerate injuries due to the lowest density of insect pests where economic loss is very negligible, and it is known as the economic injury level, or EIL (Stern et al., 1959). However, pest management strategies must be applied earlier to EIL to avoid economic losses, and this borderline is known as the Economic Threshold (ET; Pedigo et al., 1986). Modern IPM strategies utilize the ET levels to determine the application of pesticides. Modern IPM includes a suitable combination of various pest management strategies such as the cultivation of resistant plants, the application of biological control agents, and biotechnology methods (Kogan, 1998). Each of these provides differential results in pest management, but their collective effects may drastically reduce yield losses (Dara, 2019). Various crops involve diverse protocols of IPM. The severity of synthetic pesticides toward the environment and the lower viability of plant-based products limit the effectiveness of the cultural practices. Therefore, there is a need for novel strategies to eradicate the pest population without harming the ecological balance.

### 3.1. Symbionts as a novel tool for IPM

The symbiotic microbiome has a wide array of functions, such as increasing the survival rate to achieve a longer lifespan of the host insects, the fecundity rate, etc. (Blaser, 2014; Marchesi and Ravel, 2015; Yong, 2016; Douglas, 2018). The microbiome of insect pests has increased their survival rates by providing a variety of benefits to their invasiveness. These traits could be targeted to reduce the insect pest population. In the Tsetse fly, antibiotics such as tetracycline and penicillin have been introduced *via* ingestion to eliminate the symbiotic microflora. Ingestion of these antibiotics has been shown to impart sterility to the fly by disturbing symbiont *Wigglesworthia* in the larval stages and leading to failed reproductive ability (Zhong et al., 2007).

In this way, understanding insect-symbiont interaction is very important in pest control strategies. Earlier attempts to apply various antibiotics against insect symbionts to disturb their functioning are made as shown in Table 3.

**Table 3:** Antibiotics used against the symbiotic bacteria to check insect survival.

Insect	Symbiont bacteria	Antibiotics	Effects of antibiotics	References
**Midges** *Culicoides sonorensis*	*Asaia* spp.	10 μg/mL Penicillin 10 μg/mL Streptomycin	Insects became susceptible to the Schmallenberg virus	Möhlmann et al. (2020)
*Culicoides nubeculosis*	*Asaia* spp.	10 μg/mL Penicillin 10 μg/mL Streptomycin	Insects became susceptible to the Schmallenberg virus	Möhlmann et al. (2020)
**Mealy bug** *Rhizoecus amorphophalus*	*Bacillus subtilis* *Staphylococcus gallinarum Staphylococcus saprophyticus*	30 μg/mL Cephalexin 5 μg/mL Ciprofloxacin 10 μg/mL Endrofloxaxin 5 μg/mL Cefixime	Reduced survival rates	Sreerag et al. (2014)
**Fruit fly** *Bactocera minax*	*Klebsiella Citrobacter* *Proteobacteria Firmicutes*	10 μg/mL Ciprofloxacin 200 μg/mL Piperacillin	Reduced copulation rates Reduced fecundity rates No egg production (Effect observed when fed with full diet)	Andongma et al. (2018)
**Chinch bug** *Blissus insuralis*	*Burkholderia*	Kanamycin Oxytetracycline Trimethoprim Ampicillin Penicillin G Chloramphenicol 25 μL each	Reduced survival rates	Xu et al. (2016)
**Chinch bug** *Blissus insuralis*	*Burkholderia*	Chlortetracycline 1%	Reduced fecundity	Boucias et al. (2012)

### 3.2. Techniques for insect pest control

Sterile insect techniques (SIT), paratransgenesis, and incompatible insect techniques (IIT) have been proven to be very essential approaches to insect pest control. SIT is one of the eco-friendly approaches of insect pest control. This technique is popular because of its specificity and application in eradication of insect pest over wide insects order (Dyck et al., 2021; Bourtzis and Vreysen, 2021). Many factors are responsible for the success of implementing this tool, amount of radiations, rearing methods, shipping, release method and inferior performance by released males compared to wild males. Mating to sterile male results in infertile eggs which are unable to reach embryogenesis. Subsequently over the time population of target species declines and eradication takes place (Knipling, 1979). The IIT is used to interfere with or modulate the host’s innate microbiota, through which males are made incompatible for reproduction. It has been well worked out in insects such as *Drosophila* and mosquitoes (Ridley et al., 2013; Coon et al., 2014). A study was conducted using the symbiont *Wolbachia* to combat mosquitoes and related pathogens. This symbiont manipulates the host’s reproductive system through cytoplasmic incompatibility, parthenogenesis, and feminization, thereby reducing the male population (Edenborough et al., 2021). *Rhagoletis cerasi, Ceratitis capitate*, Tsetse fly, and disease vectors such as *Culex pipens* are other examples of IIT (Laven, 1967; Zabalou et al., 2009; Abd-Alla et al., 2013).

In addition to this, the technique of paratransgenesis deals with the genetic modification of the gut microbiome to express relevant traits in insects (Aksoy et al., 2008). In this method, instead of modifying the genome of the host insect, preference is given to the change in the genome of gut microbes. *Rhodnius prolixus* is the Chagas disease vector, and studies have shown that the gut symbiont *Rhodococcus rhodnii* resides in the gut of the triatomine bug *Rhodnius prolixus*. This symbiont provides the essential nutrients to the insect (Beard et al., 2000). Further modifying these symbionts genetically and reintroducing them into the host insect has shown to reduce the insect population due to lack of sufficient nutrients (Taracena et al., 2015).

#### 3.2.1. Manipulation of gut symbionts *via* CRISPR-Cas9 mechanism

Mosquitoes’ alimentary canals are home to a complex community of symbiotic bacteria with poor species diversity (Coon et al., 2016). The vectorial capacity and host phenotype in mosquitoes’ mostly modulated by gut microbes (Weiss and Aksoy, 2011). Rigorous studies have shown that genes from symbionts are implicated in colonization of host insect gut. For instance, the genes from bacterium *Snodgrasselia alvi* are responsible for gut colonization of honey bee (Powell et al., 2016). Another research has shown that there is formation of defective biofilm and reduced bacterial colonization in bean bug, after knockout of purine biosynthesis gene from symbiont *Burkholderia* (Kim et al., 2014). The successful interaction of symbiont *Sodalis glossinidius* with its host insect tsetse fly is mediated with the protein A (ompA) of outer membrane of the bacterium where as mutation in ompA gene resulted into poor colonization of symbionts in the fly gut (Weiss et al., 2008). Taken together, these studies focus on a point that genetic elements of the symbiotic bacteria are essential for formation of biofilm and symbiotic interaction.

All above mentioned studies are based on transposon mutagenesis which requires mutant library. The expression of specific bacterial gene which is required for host microbe interaction can be downregulated with a targeted gene knockout approach which is highly desirable (Maltz et al., 2012). Recently the CRISPR/Cas9 gene editing system approach is utilized to manipulate bacterial genome but this approach is rarely used for symbionts harboring the insect gut. Deciphering the role of microbial genes in the host insect and knocking them out will be a better approach to carry out insect pest control *via* paratransgenesis (Sander and Joung, 2014; Selle and Barrangou, 2015; Barrangou and van Pijkeren, 2016). Use of plasmid or transposon to manipulate symbionts of insect pest have been used in paratransgenesis but use of CRISPR/Cas9 gene editing method to integrate site specific transgenes into the symbiont genome will have great potential in paratransgenesis (Wilke and Marrelli, 2015; Arora and Douglas, 2017). For instance, the symbiont *Cedecea neteri* is required for the production of biofilm in the gut of host *Aedes aegypti.* Hegde et al., 2019 have carried out the CRISPR/Cas9 genome editing experiments by using Scarless Cas9 Assisted Recombineering (no-SCAR) strategy to disable the expression of ompA gene of the symbiont *Cedecea neteri* resulting into poor biofilm formation. Thus CRISPR/Cas9 gene editingtool can be an effective tool for genomic manipulation of symbionts of insect pest.

### 3.3. RNAi: Symbiont mediated tool for insect pest control

The path-breaking research of injecting dsRNA, specific for the target gene, paved the way for RNA dependent gene silencing methods in *Caenorhabditis elegans* (Tabara et al., 1999; Montgomery, 2004) Gene knockdown by RNAi is elucidated in plants, fungi and nematodes suggesting an evolutionary conservation of RNAi pathways among these organisms (Tabara et al., 1999; Lee and Ambros, 2001; Schott et al., 2005). While studying these pathways in insects, despite having different effector molecules, the pathways of internal gene regulation, protection from transposons and antiviral defensive system are very similar to these organisms (Yang et al., 2020). RNA sequence-based research has been utilized for achieving best measures of insect pest management since last decade (Joga et al., 2016). Several research groups have established the mechanism of RNAi *via* hairpin RNA (hp RNA), artificial microRNA and dsRNA, which can be synthesized and designed artificially to downregulate expression of specific genes from target insect species. Having high specificity in insect pest control, RNAi based pest control method faces several limitations such as high production cost, technical lacunas and public approval for GM plants. To overcome these challenges delivery of dsRNA can be carried out *via* microbes. Nymphal mortality has been observed in beetle, *Henosepilachna vigintiocto punctata* after exposure to leaves coated with *Snf7*gene-specific dsRNA synthesized by bacteria (Lü et al., 2019). These studies have proved the prominent role of RNAi in IPM, but presence of nucleases in insect gut and impact of RNAi machinery are the major obstacle to carry out successful RNAi experiments (Whyard et al., 2009).

Nevertheless, there are chances of molecular crosstalk between gut symbionts and injected dsRNA as observed in gut bacteria of beetle *Plagiodera versicolora*. While studying the lethality by ingested dsRNA, it is investigated that dsRNA degradation in insect body resulted into dysbiosis of symbionts, and the degradation products accelerated the growth of commensal bacterium *Pseudomonas putida* changing its status to crucial activator of the mortality of *Plagiodera versicolora* larva (Xu et al., 2021). Similarly, aphids being notorious pest of many crops, variety of control measures are applied to get rid of this pest. Application of RNAi proved very effective against various aphids. Despite having high specificity in this approach, the cost of its chemical synthesis and availability of dsRNA is very low. To overcome these problems, symbiont mediated RNAi (SMR) is employed where dsRNA is synthesized by engineered gut symbionts. Aphids harbor *Buchnera aphidicola* and many other facultative symbionts which are transmitted *via* vertical and horizontal transport and have wide range of hosts. These symbionts lack the genes for biosynthesis of cell surface components and have highly reduced genome, but symbiont *Candidatus Serratia symbiotica* is the first facultative endosymbiont cultured on axenic media which became useful in successful implementation of SMR. Targeting specific gene from host insect *via* Symbiont mediated RNAi is demonstrated in various insect pests and it may pave the way for novel approach in controlling insect pests (Li T. et al., 2022).

## 4. Future prespective and challenges

Cultivation of genetically modified crops has increased annually, leading to a decrease in the use of pesticides, yet there are numerous problems associated with their approval due to a lack of safety issues regarding human health. Further, the functionality of biopesticides is slow as compared to chemical pesticides, therefore insect pest control methods with faster application are needed. The microbiome of the insect gut plays a crucial role in shaping the host insect’s physiology. Provision of nutrients, modulation of the signaling pathway to trigger innate immunity, protection from plant defence and predators, breakdown of harmful pesticides ingested by the insect host, all these physiological aspects are important to study as they are influenced by gut symbionts in the insect body. The modulation of the gut microbiota is crucial for the development of novel tools in pest control methods. Studies have shown that alteration in gut microbes leads to reduced insect populations in mosquitoes. The evolution of insect pest control methods from the application of pesticides to biopesticides and now the manipulation of the gut microbiome *via* CRISPR/Cas9, RNAi, SIT, IIT and antimicrobials is considered as historic breakthrough. Methods such as the sterile insect technique coupled with the incompatible insect technique have proven crucial in pest control studies. The advent of metagenomics and transcriptomics paved the way to identify the precise role of these symbionts so that one can target the microbiome to impede the development of the paricular host. The information gathered *via* these techniques would allow us to explore different ways of exploiting the metabolism of symbionts for pest control.

However, despite their high specificity, the new molecular tools have certain gaps. Sterile insect techniques irradiate male insects, but it requires a huge population of male insects to compete with wild insects. It is very difficult to get a generation full of male insects which is the major drawback of these sterile insect techniques. In addition, the cost of these techniques is very expensive and sometimes radiations can lead to a higher rate in insects. This renders the sterile insect technique unsuitable for implementation in the field.

In addition, RNAi technology has established itself as a successful tool in genomics and its use in insect pest control. The uptake of dsRNA by insect pests, its stability, and delivery are the main concerns when applying this approach. The genes used in RNAi are not ubiquitous in insect pests. Till the date it is unclear which forces are responsible for sending silencing signals. Understanding the detailed mechanism of RNAi is still the matter of investigation and improvements in its delivery will be inventive breakthrough in the coming years with higher specificity. Effective utilization of RNAi will restore the population of natural enemies and beneficial flora of field. The large-scale production of dsRNA and its application in the field is a cumbersome and expensive process, so there is a need for low-cost production methods such as the production of dsRNA by bacteria and the modification in synthetic nucleoside triphosphates.

CRISPR/Cas9 based pest control mechanism is appreciated as one of the top 10 insect pest control methods by Science Magazine in 2013.Since this method is most elegant and affordable there are still lacunas in its application like non target effects, inefficient routes to its delivery and drawbacks in genome editing tools. CRISPR/Cas9 based methods can revolutionize the agriculture sector when these drawbacks get overcome by inclusion of pest control strategies like SIT or IIT with CRISPR/Cas9.Transcription regulation by implementing CRISPR/Cas9 will be promising approach for pest control.

Increased human population and migration, recent environmental changes, global business and trade strategies, advanced agricultural practices, and other factors have all contributed to the threat of invasive insect pests. Extensive use of chemical pesticides has been proven to be an essential and immediate remedy for controlling insect pests. However, because of their negative effects on ecosystems and human health, there is a high demand for a novel eco-friendly pest control technique. With the advent of advanced techniques, the myriad beneficial roles of the symbiont microorganisms toward their insect hosts are well documented. Complete or partial knockdown of symbiont microbes may result in deprived nutrition, hampered immunity, weakened plant defence mechanisms, and increased susceptibility to pathogens, parasites, predators, etc. Suppression of symbionts will hamper the ability of host insects to perform the most crucial activities, so it may lead to the partial or complete removal of insect pests from fields. Thus, utilizing various strategies such as sterile insect techniques, CRISPR/Cas9, RNAi or complete removal of gut microbes might be the most appropriate potential approaches toward integrated pest management. For decades, only a handful of microbes have been in use for pest management, inventing the methods of biocontrol. Detailed studies of these symbiont microbes of insect pests and their manipulation can open up a new era in the pest control strategy. Development of integrated pest management system with zero side effects on non-target species can guarantee agricultural improvement and food safety.

## Author contributions

PSR, SB, and PR: conceptualization. PSR, DJ, and SB: writing–original draft. PR, KK, SG, AA, DJ, and SB: review and editing. All authors contributed to the article and approved the submitted version.

## Conflict of interest

The authors declare that the research was conducted in the absence of any commercial or financial relationships that could be construed as a potential conflict of interest.

## Publisher’s note

All claims expressed in this article are solely those of the authors and do not necessarily represent those of their affiliated organizations, or those of the publisher, the editors and the reviewers. Any product that may be evaluated in this article, or claim that may be made by its manufacturer, is not guaranteed or endorsed by the publisher.

## Abbreviations

AMP: Antimicrobial peptide
APSE: *Acyrthosiphon pisum* secondary endosymbiont
CRISPER: Clustered regularly interspaced short palindromic repeats
FAO: Food and agriculture organization of the United States
GST: Glutathione sulfur transferase
IIT: Incompatible Insect Technique
IPM: Integrated pest management
JA: Jasmonic acid
MEP: Methyl Parathion
NADPH: Nicotinamide adenine dinucleotide phosphate
PBE: Plant-associated beneficial bacteria
RNAi: RNA interference
ROS: Reactive oxygen species
SA: Salicylic acid
SBE: Stink bug-associated beneficial bacteria
SIT: Sterile insect techniques
SOPE: *Sitophilus oryzea* principal endosymbiont
